# Implementation and Quality Improvement Strategies in Assisted Living: A Scoping Review

**DOI:** 10.1093/geroni/igaf122.2395

**Published:** 2025-12-31

**Authors:** Cameron Ulmer, Jennifer Leeman, Sheryl Zimmerman, Mark Toles

**Affiliations:** The University of North Carolina at Chapel Hill, Chapel Hill, North Carolina, United States; The University of North Carolina at Chapel Hill, Chapel Hill, North Carolina, United States; University of North Carolina at Chapel Hill, Chapel Hill, North Carolina, United States; The University of North Carolina at Chapel Hill, Chapel Hill, North Carolina, United States

## Abstract

Gaps persist in the quality of care in assisted living (AL), yet little is known about the best approaches for closing these gaps. Guided by Proctor’s Framework for Implementation Research, this scoping review examined studies of quality improvement strategies and outcomes in AL. In collaboration with a health sciences librarian, we systematically searched PubMed, CINAHL, and Scopus to identify English-language, empirical, peer-reviewed, quality improvement and implementation studies in AL published before May 2023. Of 204 studies identified, 9 met inclusion criteria. The clinical focus of papers included behavioral expressions of persons with dementia (n = 2), activities of daily living (n = 2), and antibiotic stewardship (n = 1), among others. Strategies used to implement new clinical practices included staff training (n = 9), the addition of new staff and/or new tools (n = 8), and rapid cycles of improvement (n = 5). Studies reported outcomes related to both feasibility (n = 7), (e.g., intervention adherence, acceptability), and intervention effectiveness (n = 7), (e.g., pre- and post-intervention testing, objective observation). While sample sizes tended to be small, most interventions demonstrated preliminary evidence of effectiveness (n = 6), with only one (n = 1) reporting statistically significant outcomes. Common gaps in study reports included estimates of statistical power, details regarding missing data and unintended consequences, and the cost of the interventions. In summary, few implementation or quality improvement studies have been published on the AL setting and more empirical research is necessary to understand strategies for closing gaps in AL care quality.

